# Web-Based Versus Traditional Paper Questionnaires: A Mixed-Mode Survey With a Nordic Perspective

**DOI:** 10.2196/jmir.2595

**Published:** 2013-08-26

**Authors:** Lena Hohwü, Heidi Lyshol, Mika Gissler, Stefan Hrafn Jonsson, Max Petzold, Carsten Obel

**Affiliations:** ^1^Department of Public HealthSection for General PracticeAarhus UniversityAarhus CDenmark; ^2^Department of Health StatisticsNorwegian Institute of Public HealthOsloNorway; ^3^THL National Institute for Health and WelfareHelsinkiFinland; ^4^NHV Nordic School of Public HealthGothenburgSweden; ^5^University of Iceland and The Directorate of HealthReykjavikIceland; ^6^Sahlgrenska AcademyCentre for Applied BiostatisticsUniversity of GothenburgGothenburgSweden

**Keywords:** mixed-mode survey, patient participation rate, Web-based, paper, questionnaires, nonmonetary incentive

## Abstract

**Background:**

Survey response rates have been declining over the past decade. The more widespread use of the Internet and Web-based technologies among potential health survey participants suggests that Web-based questionnaires may be an alternative to paper questionnaires in future epidemiological studies.

**Objective:**

To compare response rates in a population of parents by using 4 different modes of data collection for a questionnaire survey of which 1 involved a nonmonetary incentive.

**Methods:**

A random sample of 3148 parents of Danish children aged 2-17 years were invited to participate in the Danish part of the NordChild 2011 survey on their children’s health and welfare. NordChild was conducted in 1984 and 1996 in collaboration with Finland, Iceland, Norway, and Sweden using mailed paper questionnaires only. In 2011, all countries used conventional paper versions only except Denmark where the parents were randomized into 4 groups: (1) 789 received a paper questionnaire only (paper), (2) 786 received the paper questionnaire and a log-in code to the Web-based questionnaire (paper/Web), (3) 787 received a log-in code to the Web-based questionnaire (Web), and (4) 786 received log-in details to the Web-based questionnaire and were given an incentive consisting of a chance to win a tablet computer (Web/tablet). In connection with the first reminder, the nonresponders in the paper, paper/Web, and Web groups were also present with the opportunity to win a tablet computer as a means of motivation. Descriptive analysis was performed using chi-square tests. Odds ratios were used to estimate differences in response rates between the 4 modes.

**Results:**

In 2011, 1704 of 3148 (54.13%) respondents answered the Danish questionnaire. The highest response rate was with the paper mode (n=443, 56.2%). The other groups had similar response rates: paper/Web (n=422, 53.7%), Web (n=420, 53.4%), and Web/tablet (n=419, 53.3%) modes. Compared to the paper mode, the odds for response rate in the paper/Web decreased by 9% (OR 0.91, 95% CI 0.74-1.10) and by 11% (OR 0.89, 95% CI 0.73-1.09) in the Web and Web/tablet modes. The total number of responders for NordChild declined from 10,291 of 15,339 (67.09%) in 1984 and 10,667 of 15,254 (69.93%) in 1996 to 7805 of 15,945 (48.95%) in 2011 with similar declines in all 5 Nordic countries.

**Conclusions:**

Web-based questionnaires could replace traditional paper questionnaires with minor effects on response rates and lower costs. The increasing effect on the response rate on participants replying for a nonmonetary incentive could only be estimated within the 2 Web-based questionnaire modes before the first reminder. Alternative platforms to reach higher participation rates in population surveys should reflect the development of electronic devices and the ways in which the population primarily accesses the Internet.

## Introduction

### Background

Epidemiological studies have seen response rates decline by approximately 1% per year in many countries in recent years [1-3]. In general, a low response rate may increase the risk that respondents differ systematically from nonrespondents, in which case the results may not be representative of the study population [4]. This is not a serious problem in studies testing a specific causal hypothesis [3]. However, differential selection of respondents may seriously bias the results in descriptive and cross-sectional studies, and it is desirable to develop methods to maximize the response rate to achieve optimum external validity.

### Response Rates in Paper- and Web-Based Questionnaires

The paper questionnaire has been the epidemiological mode of choice for collecting survey data so far, but with the increasing use of the Internet, Web-based questionnaires may be an obvious alternative. Web-based questionnaires have been shown to lower data collection costs [5,6], which is attractive especially in large population-based surveys.

The response rates in most studies so far, however, have been reported to be lower in Web-based questionnaires than in paper-based questionnaires [5,7], but the opposite has also been reported [8]. In a questionnaire survey on patients’ experiences with breast cancer care, no significant difference was observed between the response rates of a mailed paper questionnaire only (64.0%) and an online questionnaire followed by a paper reminder (60.5%) [5]. A Danish questionnaire survey reported a statistically significantly higher total response rate in a paper-and-pencil group (76.5%) than in a group with access to the questionnaire via log-on to the Internet (64.2%) [7]. A study comparing mixed-mode (paper or online) and Web-based questionnaires exploring fertility issues among female childhood cancer survivors found a 6% higher participation rate in the Web-based mode (89%) than in the mixed mode (83%) [8].

### Incentives

A number of previous studies suggest that monetary or lottery incentives increase response rates and that such incentives may be used to raise participant representativeness [4,9-12]. They showed an improvement in the response rate of 2 percentage points by the use of a US $5 versus a US $2 incentive, and that use of a £10 gift voucher gave 45% higher odds of responding than use of no incentive. The respondent in Web-based questionnaires may simply skip difficult items, which increases the risk of incompletely filled-in questionnaires, although this problem has also been noticed in paper questionnaires. The inclination to skip items and the resulting lower rate of fully completed questionnaires has been seen predominantly in Web-based versions, but the problem has also been noticed in paper questionnaires [5,13]. However, an incentive both reduces item nonresponse and improves participation among participants with lower education levels [14].

### Access to the Internet

The rapid growth in access to the Internet in developed countries has decreased the coverage differential between paper- and Web-based questionnaires and, thus, the risk of selection bias by using the Internet for questionnaire purposes [1]. In 2012, 99% of Danish couples with children had access to a computer and the Internet at home compared with 86% of all Danish families in general [15]. In Denmark, there is free public access to the Internet at all libraries, which ensures 100% access to the Internet for the whole population.

The questionnaire survey “Health and welfare among children and young people in the Nordic countries” (NordChild) was previously conducted in 1984 (NordChild1984) and 1996 (NordChild1996). The response rate increased from 67.09% in 1984 to 69.93% in 1996 [16]. Because of the general tendency observed in other studies [1,3], we expected the NordChild 2011 attrition to be considerably lower. In light of the advancement of Web-based technology and of its growing reliability, the Internet may be considered an obvious means for questionnaire data collection or at least a mode supplementary to the traditional paper questionnaire, not least because of its comparative advantages in terms of lower costs and simpler logistics [5,6]. Adding an incentive to the Internet-based questionnaire is thought to increase the response rate and to improve the quality of the answers [9]. To our knowledge, little is known about whether the presence of a single chance of receiving a nonmonetary incentive improves the response rate. The aim of the present paper is to compare response rates in a questionnaire survey by using 4 different modes of data collection, including 1 with a nonmonetary incentive.

## Methods

### The Danish Survey

A total of 3200 parents of children aged 2 to 17 years living in Denmark in 2011 were randomly selected to participate in the third NordChild questionnaire survey. There were 200 children in each of the 16 year groups between 2 and 17 years of age: 100 girls and 100 boys. The random selection of 1 child per family was managed by the Danish National Board of Health. All citizens in Denmark have a unique 10-digit personal identification number. The parents and their addresses were identified through the Danish Civil Registration System [17], and persons who had prohibited the use of their addresses for marketing purposes or scientific studies were excluded before the selection [18]; in 2009, this group accounted for 12.8% of the Danish population [19]. The Danish questionnaire consisted of 73 questions with subquestions, and the paper version was 28 pages long. The study was approved by the Danish National Board of Health. For the analysis, the unique 10-digit personal identification number was encrypted.

A total of 52 invited respondents were excluded in the analysis; 5 announced that they did not wish to participate in the survey, 1 because of difficulties understanding written Danish, and 46 because the child had turned 18 years of age after the sample was selected and before the questionnaire was mailed.

The 3200 invited children were randomly allocated into 4 equal modes, and after exclusion they (N=3148) were allocated as following: (1) 789 received a paper questionnaire only (paper), (2) 786 received the paper questionnaire as well as a log-in code to the Web-based questionnaire (paper/Web), (3) 787 received log-in information to the Web-based questionnaire only (Web), and (4) 786 received log-in details to the Web-based questionnaire plus an incentive consisting of a chance to win a tablet computer (Web/tablet). Overall, the paper and paper/Web groups were categorized as paper versions of the questionnaire and the Web and Web/tablet as Web-based questionnaires. We choose to allocate the incentive in the Web-based questionnaires because the Web and Web/tablet modes are comparable. The Web-based questionnaire was a multipage design using SurveyXact [20] and had the same questions as the paper version did. The respondents of the Web-based questionnaire could answer the questions in several rounds, and submit it after the last question. A 12-character log-in code to the Web-based questionnaire had to be keyed in every time if it was not completed in a single round.

The data collection ran over a 4-month period, starting on June 6 and closing on October 6, 2011. All 3200 were invited by mail. Two reminders were mailed to all those who had not responded 4 and 12 weeks after the invitation to participate, on July 6 and August 31, respectively. Nonresponders at both the first and the second reminder were offered online participation only, but could receive a paper version of the questionnaire if they requested it. In connection with the first reminder, the nonresponders in the paper, paper/Web, and Web groups also were given the opportunity to win a tablet computer as means of motivation. In all, 2 tablet computers were distributed in the data collection period; the first to the responders in the Web/tablet mode, and the second to the responders who responded after the initial reminder in the paper, paper/Web, and Web modes.

Costs per responder were estimated. The estimation included distribution and collection of the questionnaires: printing the paper questionnaire, printing the 2 reminders to nonresponders, salary for student workers to pack all the invitations and scanning of returned paper questionnaire, postage (the invitations, prepaid envelope of returned questionnaires, and the 2 reminders to the nonresponders), 2 tablet computers (the cost of the first divided by all 800 in the Web/tablet mode, the cost of the second divided between the nonresponders in the paper, paper/Web, and Web mode), and layout of a website for the online questionnaire. Packing of the 2 reminders and programming of the questionnaire involved no extra cost that had to be covered by the general budget at the Department of Public Health, Aarhus University. Costs in Euro (€) are stated in 2011 prices.

The Danish 2011 survey was registered at The Danish Protection Agency (Journal number: 2011-41-6230).

### The Common Nordic Survey

The NordChild survey was first conducted in 1984 and then in 1996 in all the Nordic countries: Finland, Iceland, Norway, Sweden, and Denmark [16]. In each of the years the survey took place, approximately 3000 randomly selected parents of children between the ages of 2 and 17 years were invited to participate in each of the Nordic countries. In total, 46,590 children were invited during the 3 periods. Except during the Danish 2011 survey, all invited respondents were asked to fill out a paper version of the questionnaire. The questionnaire largely consisted of the same questions each time the survey was conducted. An additional 13 questions were added in the 2011 survey; thus, the questionnaires distributed in 2011 in the 5 Nordic countries consisted of 73 questions. Information on numbers of reminders, the use of an incentive, and the final response rate in each country were collected from the national contact persons.

### Statistical Analysis

Descriptive analysis was performed using chi-square tests (χ^2^). In the Danish 2011 survey, the responders and nonresponders were tested for each of the following descriptive characteristics, stratified by the 4 different modes: gender, age of the child, mother’s and father’s age, and urbanity of residence. The urbanity at municipality level was divided into 2 categories: more than and less than 100,000 citizens (the capital area of Copenhagen, Aarhus, Aalborg, Odense, Esbjerg, and Vejle in the top category). An odds ratio with 95% confidence interval (CI) estimated differences in the response rates between the 4 modes. In the analysis, the proportions of the included participants in each mode were based on initial invitation. The overall response rate for NordChild where tested in each of the years 1984, 1996, and 2011. The statistical analyses where performed in Stata version 11 (StataCorp LP, College Station, TX, USA). A *P* value <.05 was considered statistically significant.

## Results

### The Danish Survey

**Figure 1:**
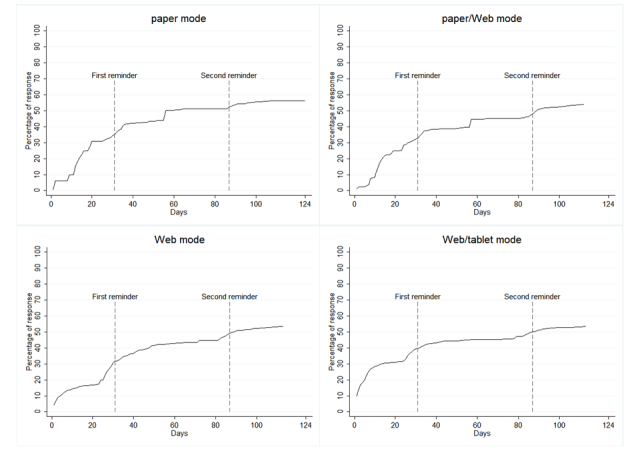
Percentage of received questionnaires by days for the paper (paper-based questionnaire only), paper/Web (paper- and/or Web-based questionnaire), Web (Web-based questionnaire only), and Web/tablet (Web-based questionnaire with tablet computer incentive within 14 days of invitation) modes in the Danish 2011 NordChild questionnaire survey.

Overall, 1704 of 3148 (54.13%) respondents answered the questionnaire. The percentages of received questionnaires by days are shown in Figure 1. The highest response was seen in the Web/tablet mode in the first 20 days. The highest final response rate was obtained in the paper mode in which 443 of 789 (56.2%) responded. A similar response rate was seen in the paper/Web, Web, and Web/tablet modes: 422 of 786 (53.7%), 420 of 787 (53.4%), and 419 of 786 (53.3%) returned the questionnaire, respectively. The overall response rate before first reminder was 34.75% (1094/3148); stratified by modes, it was 35.0% (276/789) in the paper mode, 32.8% (258/786) in the paper/Web mode, 31.6% (249/787) in the Web mode, and 39.6% (311/786) in the Web/tablet mode. For Web and Web/tablet modes that were comparable except for an incentive, the response rate was statistically significant higher in the Web/tablet mode (*P*=.001) before the first reminder. The overall response rate before second reminder was 49.78% (1567/3148); stratified by modes, it was 52.3% (413/789) in the paper mode, 47.8% (376/786) in the paper/Web mode, 48.9% (385/787) in the Web mode, and 50.0% (393/786) in the Web/tablet mode.

In connection with the first reminder, the nonresponders in the paper, paper/Web, and Web modes were also given the opportunity to win a tablet computer as a means of motivation.

The characteristics of the responders and nonresponders in the Danish 2011 survey are given in Table 1. For the age groups of the children, the highest response rates were among parents of children aged 2 to 5 years in the paper/Web (106/422, 56.7%), Web (114/420, 60.6%), and Web/tablet (100/419, 55.3%) modes. For the paper mode, the highest response rate was seen in parents of children aged 10 to 13 years (122/443, 62.6%). There were a higher number of responders from intermediate-sized urban areas outside the largest cities in Denmark.

Compared to the paper mode, the odds for response rate in the paper/Web mode decreased by 9% (OR 0.91, 95% CI 0.74-1.10) and by 11% (OR 0.89, 95% CI 0.73-1.09) in the Web and Web/tablet mode (Table 2). Of the 422 responders in the paper/Web mode, 281 (66.6%) preferred to answer the questionnaire by paper and 141 (33.4%) preferred the Web-based version (*P*<.001). Of responders who answered the first question in the questionnaire, 413 of 419 (98.6%) responders in the paper/Web mode also answered the last question. In the paper mode, these numbers were 427 of 442 (96.7%), 387 of 416 (93.0%) in the Web/tablet mode, and 377 of 416 (90.6%) in the Web mode.

The costs per responder for distribution and collection of the paper questionnaires and the Web-based questionnaires are shown in Table 3. The cost of the paper questionnaires (€9.02) was twice that of the Web-based questionnaires (€4.55) with the postage as the single most costly item.

### The Common Nordic Survey

In the common Nordic survey, the total numbers of responders declined from 10,291 of 15,339 (67.09%) in 1984 and 10,667 of 15,254 (69.93%) in 1996 to 7805 of 15,945 (48.95%) in 2011 (Table 4). For the previous years, the response rates for the NordChild 2011 were statistically significantly different across the participating countries (*P*<.001). An 8% difference was observed between the lowest rate in Sweden (1461/3197, 45.70%) and the highest in Denmark (1704/3148, 54.13%).

**Table 1:** Characteristics of the responders in the Danish part of the 2011 NordChild questionnaire survey.

Characteristics		Mode^a^											
		Paper (n=789)			Paper/Web (n=786)			Web (n=787)			Web/tablet (n=786)		
		Yes	No	*P* ^b^	Yes	No	*P* ^b^	Yes	No	*P* ^b^	Yes	No	*P* ^b^
Responding, n		443	346		422	364		420	367		419	367	
**Gender, n (%)**													
	Male	234 (59.1)	162 (40.9)		198 (50.0)	198 (50.0)		203 (51.7)	190 (48.4)		209 (53.3)	183 (46.7)	
	Female	209 (52.2)	184 (46.8)	.09	224 (57.4)	166 (42.6)	.04	217 (55.1)	117 (44.9)	.34	210 (53.3)	184 (46.7)	.99
**Age of child (years), n (%)**													
	2-5	109 (57.1)	82 (42.9)		106 (56.7)	81 (43.3)		114 (60.6)	74 (39.4)		100 (55.3)	81 (44.7)	
	6-9	111 (56.9)	84 (43.1)		111 (54.7)	92 (45.3)		108 (54.6)	90 (45.4)		103 (50.0)	103 (50.09)	
	10-13	122 (62.6)	73 (37.4)		101 (50.8)	98 (49.2)		100 (50.3)	99 (49.7)		108 (53.7)	93 (46.3)	
	14-17	101 (48.6)	107 (51.4)	.04	104 (52.8)	93 (47.2)	.68	98 (48.5)	104 (51.5)	.08	108 (54.6)	90 (45.4)	.73
**Maternal age (years), n (%)**													
	<35	71 (50.3)	70 (49.7)		71 (50.7)	69 (49.3)		66 (52.8)	59 (47.2)		57 (41.9)	79 (58.1)	
	35-44	265 (57.5)	196 (42.5)		237 (53.4)	207 (46.6)		246 (53.4)	215 (46.6)		248 (54.0)	211 (46.0)	
	≥45	105 (58.5)	75 (41.7)	.35	112 (56.6)	86 (43.5)	.56	104 (53.1)	92 (46.9)	.99	113 (60.1)	75 (39.9)	.01
**Paternal age (years), n (%)**													
	<35	41 (52.6)	37 (47.4)		45 (51.1)	43 (48.9)		37 (51.4)	35 (46.6)		40 (51.3)	38 (48.7)	
	35-44	235 (57.3)	175 (42.7)		203 (55.5)	163 (44.5)		226 (54.1)	192 (45.9)		194 (50.1)	193 (49.9)	
	≥45	160 (55.9)	126 (44.1)	.73	168 (53.5)	146 (46.5)	.73	148 (52.9)	132 (47.1)	.89	175 (59.5)	119 (40.5)	.05
**Urban area, n (%)**													
	≤100,000	303 (58.5)	215 (41.5)		266 (53.9)	228 (46.1)		290 (55.3)	234 (44.7)		280 (54.1)	238 (45.9)	
	>100,000	140 (51.7)	131 (48.3)	.07	156 (53.4)	136 (46.6)	.91	130 (49.4)	133 (50.6)	.12	139 (51.9)	129 (48.1)	.56

^a^Paper: paper version of questionnaire, paper/Web: paper and/or Web-based questionnaire, Web: Web-based questionnaire, Web/tablet: Web-based questionnaire with opportunity to win a tablet computer within the first 14 days after invitation.

^b^Between responders (Yes) and nonresponders (No).

**Table 2:** The odds ratio of response rate by the 4 different modes in the Danish 2011 NordChild questionnaire survey.

Mode^a^	Odds ratio (95% CI)
Paper	1 (ref)
Paper/Web	0.91 (0.74, 1.10)
Web	0.89 (0.73, 1.09)
Web/tablet	0.89 (0.73, 1.09)

^a^Paper: paper version of questionnaire, paper/Web: paper and/or Web-based questionnaire, Web: Web-based questionnaire, Web/tablet: Web-based questionnaire with opportunity to win a tablet computer within the first 14 days after invitation.

**Table 3:** Costs (2011 prices) per respondent for distribution and collection of the Danish 2011 NordChild questionnaire survey.

Cost components		Costs (€)^a^			
		Paper	Web	Paper-Web	Paper/Web
Questionnaire, print		1.39	—	—	—
**Envelope**					
	Invitation	0.29	0.10	0.19	2.9
	2× reminders	0.20	0.20	0.00	1.0
**Student worker**					
	Packing, invitation	0.12	0.05	0.07	2.4
	Scanning	0.86	—	—	-
**Postage**					
	Invitation	2.49	0.74	1.75	3.4
	Prepaid envelope	1.55	—	—	-
	2× reminders	1.48	1.48	0.00	1.0
**Tablet computer**					
	First distribution	—	0.76	—	-
	Second distribution	0.40	0.31	0.09	1.3
**Print of reminder**					
	First	0.04	0.04	0.00	1.0
	Second	0.20	0.20	0.00	1.0
	Layout website	—	0.67	—	-
Total per responder		9.02	4.55		
Measurement				4.47	2.0

^a^Paper: paper questionnaires (paper and paper/Web), Web: Web-based questionnaires (Web and Web/tablet), paper–Web: costs of paper questionnaires minus costs of Web questionnaires, paper/Web: ratio of costs of paper questionnaires and costs of Web questionnaires, —: no cost.

**Table 4:** The response rate and description of data collection for the 2011 NordChild questionnaire survey.

Data collection		Participating countries					Total	*P* value^a^
		Denmark	Finland	Iceland	Norway	Sweden		
**Responders, n (%)**								
	1984	2219 (73.65)	2705 (83.21)	1577 (59.51)	1856 (55.80)	1934 (62.41)	10,291 (67.09)	<.001
	1996	2169 (68.64)	2384 (79.49)	2048 (68.11)	1936 (64.51)	2130 (69.00)	10,667 (69.93)	<.001
	2011	1704 (54.13)	1538 (48.06)	1521 (47.53)	1581 (49.41)	1461 (45.70)	7805 (48.95)	<.001
**Mode of questionnaire** ^b^								
	1984	PQ	PQ	PQ	PQ	PQ		
	1996	PQ	PQ	PQ	PQ	PQ		
	2011	PQ & WBQ^c^	PQ	PQ	PQ	PQ		
**Number of reminders**								
	1984	2	2	2	2	2		
	1996	2	2	2	2	2		
	2011	2^d^	1	1^e^	2	2		
**Incentive**								
	1984	No	No	No	No	No		
	1996	No	No	No	No	No		
	2011	Yes^f^	No	No	No	No		

^a^ *P* value for difference in response rates.

^b^PQ: paper questionnaires, WBQ: Web-based questionnaires.

^c^Distribution: 800 paper only, 800 paper or Web-based, 1600 Web-based only.

^d^Log-on code to the Web-based questionnaire.

^e^Thank-you note to all invited participants.

^f^Tablet computer (2 incentives during different points of time).

## Discussion

### Principal Results

The main findings of this study are that 1704 of 3148 (54.13%) invited respondents answered the Danish questionnaire. In the 4 modes, the response rate was slightly higher, but not statistically significant, in the paper (443/789, 56.2%) mode than in the paper/Web (422/786, 53.7%), Web (420 /787, 53.3%), and the Web/tablet (419/786, 53.3%) modes. The failure of a nonmonetary incentive to affect response rates in all 4 modes investigated in the Danish part of the study may partly be because of a lack of a comparable reference group. The Web-based questionnaire mode carried fewer costs than the other modes. Overall, 7805 of 15,945 (48.95%) participated in the NordChild 2011 with statistically significant differences in response rates between the countries (*P*<.001).

### Comparison With Previous Studies

The slightly higher final response rate in the paper mode than in the other modes confirms findings reported in some other studies [5,7], but not in all [8]. The latter study reported a higher response rate in the Web-based questionnaire than in the paper-based version, but its study population was younger (mean age 30 years) than the population in our study in which the mean parental age was approximately 40 years. In the studies which reported the highest response rates for the paper version, the study populations’ age range was either equivalent to the age span of our population (30-60 years) [7] or it was slightly higher (mean age 55 years) [5].

The study with the younger study population compared a Web-based version with a mixed-mode questionnaire [8]. It showed that among participants who had a choice between a paper-based and a Web-based questionnaire, most preferred to answer the questionnaire using the paper version (83%), whereas only 17% answered the questionnaire online [8]. This inclination was also seen in the present study in which two-thirds of the respondents in the paper/Web mode returned a paper version of the questionnaire.

Questionnaire participants’ propensity to respond is likely shaped by the relevance they ascribe to the questionnaire. Hence, the participants in previous studies [5,7,8] were all current or previous victims of disease or a benign abnormality and their high response rates (60%-89%) may testify to the relevance of the questionnaires to their current or previous health. It is possible that a survey on health and welfare among children and young people is of little concern to some parents; therefore, we may have overvalued it as a clear topic and motivation to participate in our study.

Assessing the effect of the chance that a single responder would receive a large nonmonetary incentive was only possible for the 2 comparable modes, Web and Web/tablet, in the period from the initial invitation to the first reminder. Therefore, the present study is only contributing with information to the lacking knowledge about the effect on the response rates of offering a large, nonmonetary incentive in a Web-based questionnaire survey in the first month, conducted among a population familiar with and enjoying largely unrestrained access to the Internet. The increase in received questionnaires between the first and the second reminders for the paper and Web mode of 17.3% and 15.0% for the paper/Web mode partly may be ascribed to the incentive and partly because of the effect of being reminded about the questionnaire. An alternative to the incentive could have been to offer to communicate the study results to the participants, but previous studies have shown that this does not increase the response rates [9,21].

We found the costs for the paper questionnaire to be double the costs of the Web-based questionnaire, which is in-line with other studies [5,6]. The lower costs and the advantages of the Web logistics suggest that the Web-based questionnaire may be an alternative to paper questionnaires. The slightly lower response rate means that the costs per respondent may increase [1], which implies that the difference in costs between the paper and Web-based questionnaire may have been even larger if we had obtained a higher participation rate.

### Strengths and Limitations

A strength of the present study is that the invited study population is clearly representative of the whole population. Furthermore, we have access to registers and, thereby, the possibility to make follow-up studies of the children’s health and welfare by use of the unique 10-digit personal identification number. Our study also has certain limitations. The Danish data are generalized to the Nordic countries and to other countries with similar assess to the Internet, but the Nordic data may not be generalized to countries with unequal access to the Internet because of different Internet patterns of behavior. Furthermore, our data may not be extrapolated to other age groups because of possible changes in Internet patterns of behavior.

Our anticipation was not met that the Web-based questionnaires would feature the highest final response rate and that the final response rate would be highest in the Web/tablet mode, although the highest response rate before the first reminder was seen in the Web/tablet mode. This may be because of lack of a comparable reference group because all participants approached for the paper, paper/Web, Web, and Web/tablet modes were offered a chance to win a tablet computer during different points of time. It may also be because of the length of the questionnaire with 73 questions. A questionnaire of this length requires that a relatively large amount of time is spent answering the questionnaire. The length of the questionnaire was due primarily to addition of the Strength and Difficulties Questionnaire [22] to the NordChild 2011 survey, which had a considerable number of subquestions. The paper version was the slightly preferred questionnaire mode based on the difference in response rate and that most respondents in the paper/Web mode returned the paper version of the questionnaires. This preference for the paper version may be rooted in tradition and in convenience answering a paper questionnaire in several rounds. The 12-character log-in code to the Web-based questionnaire may have hindered the process of completing it in several rounds. However, there may also be a limit to how long a Web-based questionnaire can be. Yet, a certain number of questions must be asked in health questionnaire surveys to allow due analysis of the issues explored. Decreasing the number of questions in the present study would preclude comparison with the previous studies in 1984 and 1996, which would rob the NordChild survey of its potential to make comparisons over time.

We first planned to offer only 1 tablet computer to those who were invited in the Web/tablet mode. However, an overall response rate of 34.8% 1 month after the initial invitation was unacceptably low. Therefore, we offered another tablet computer to motivate the nonresponders in the paper, paper/Web, and Web mode with the mailed first reminder. The effect of this initiative may be blurred by allowing the nonresponders in the paper mode to answer the questionnaire online as well. The effect of offering an incentive should also be appraised in light of the nature of that incentive. Winning a tablet computer might simply not have been sufficiently attractive to make potential respondents engage in answering a comprehensive questionnaire because Denmark saw a significant rise in household possession of tablet or minicomputers from 9% in 2011 to 19% in 2012 (ie, during the study period) [23].

From a socioeconomic perspective, it would have been interesting to know which responders sent in their questionnaires right away, and if there were social differences in the need for motivation rooted in the hope of winning a tablet computer. Although we registered the date the questionnaires were returned, we collected no information about what had motivated the responders to answer the questionnaire.

### The Total Response Rate in the NordChild survey

The highest response rate in the NordChild 2011 survey was obtained in the Danish part. For the NordChild surveys in 1984 and 1996, the highest response rate was observed in Finland. The responders and nonresponders may differ between the Nordic countries, but response rates varied less between the Nordic countries in the NordChild 2011 survey than in the surveys in 1984 and 1996. In general, the data collection for the NordChild 2011 survey was less consistent than the data collection in 1984 and 1996. Thus, in Denmark, an online questionnaire was introduced together with an incentive, whereas 1 reminder was mailed in Iceland and Finland compared with 2 reminders in Sweden, Norway, and Denmark. Furthermore, a thank-you note was sent to all the invited respondents in Iceland, including 1 sentence as a reminder if the sampled person had not yet responded. The proportion of people who had a priori declined research participation in the form of postal surveys also varied between the Nordic countries. In Denmark, this figure exceeded 10%, whereas it was approximately 4% in Finland. In other Nordic countries, the survey was also sent to those who had banned the commercial use of their addresses. In other words, it remains unknown how much these differences biased the results.

Despite statistically significant differences in response rates, the different starting points of distribution, and the prospect of winning a tablet computer in the Danish part of the survey, the clinical response rate for the NordChild 2011 study was almost the same across the Nordic countries. The participants in the other 4 Nordic countries were unaware of the incentive in the Danish part of the NordChild 2011 survey which entails nondifferential selection bias. We believe that the low response rate observed in all the Nordic countries is more likely because of a general decrease in volunteerism, higher demands for participation, and oversurveying [1].

### Perspectives

The declining response rates in population-based surveys in general are a challenge to epidemiology. First, the overall response rate of 48.95% in the NordChild 2011 study makes it difficult to compare the results of the survey with the results of previous NordChild surveys in which the response rates were considerably higher. Second, the low participation rate could raise the question whether the responders are representative of the general population. Analyses linking data with administrative registers are needed to determine to which extent randomly selected responders represent the general population. Furthermore, information about what motivates parents to participate in a scientific study about their child’s health also needs to be elucidated to determine to which extent incentives, such as the results of the survey, should be offered to raise response levels to desired levels.

The present study suggests that Web-based questionnaires and the chance of winning a tablet computer are unlikely to solve the problem of low respondent attendance; therefore, other solutions must be considered. Like many other developed countries, the Nordic countries regularly conduct routine child health examinations from gestation to the end of school age that, together with the school setting itself, provide a platform for gathering valid and high-coverage survey information. By using a clinical meeting with the child and the mother or father (when the child is younger) could be a promising way to get more complete epidemiological data at the population level. In Denmark, attendance to these routine examinations is in the range 80% to 95% and the development of common Web-based tools with an interactive clinical component, such as Schoolhealth.eu, may be a means for monitoring the development of child health [24]. Future Web-based surveys may also benefit from the use of smartphones, which every second Dane is in possession of now [25].

### Conclusions

Web-based questionnaires could replace traditional paper questionnaires based on comparable response rates and lower costs. The increasing effect on the response rate on participants replying for a nonmonetary incentive could only be estimated within the 2 Web-based questionnaire modes before the first reminder. The difference in costs between the paper and Web-based questionnaires will favor the Web-based mode provided high response rates can be obtained. Web-based questionnaires provide an alternative to the traditional paper version; however, we need to consider alternative platforms to reach higher participation rates in such population surveys. Such alternatives should reflect the development of electronic devices and the ways in which the population primarily accesses the Internet.

## Abbreviations

NordChild: “Health and welfare among children and young people in the Nordic countries” questionnaire survey
PQ: all paper versions of the questionnaire (paper and paper/Web)
paper: paper questionnaire only
paper/Web: paper questionnaire as well as log-in code to the Web-based questionnaire
WBQ: all Web-based questionnaires (Web and Web/tablet)
Web: Web-based questionnaire only
Web/tablet: Web-based questionnaire plus an incentive consisting of a chance to win a tablet computer
